# STM sustains stem cell function in the *Arabidopsis* shoot apical meristem and controls *KNOX* gene expression independently of the transcriptional repressor AS1

**DOI:** 10.4161/psb.28934

**Published:** 2014-04-28

**Authors:** Simon Scofield, Walter Dewitte, James AH Murray

**Affiliations:** School of Biosciences; Cardiff University; Cardiff, UK

**Keywords:** stem cell, KNOX gene, meristem, cytokinin, KNOTTED, SHOOT MERISTEMLESS

## Abstract

The *Arabidopsis* *KNOX* gene *SHOOT MERISTEMLESS* (*STM*) is required for both the development and the sustained function of the shoot apical meristem (SAM) and can induce de novo meristem formation when expressed ectopically. STM acts through induction of cytokinin (CK) synthesis to inhibit cellular differentiation and additionally functions to organize undifferentiated cells into a self-sustaining meristem. STM has been shown to positively regulate the related *KNOX* genes *KNAT1/BP* and *KNAT2*, and it has been proposed that this is mediated through repression of the ARP-type transcriptional repressor ASYMMETRIC LEAVES1 (AS1). Here we investigate the role of *STM* in SAM organization, stem cell maintenance and the regulation of *KNOX* gene expression. We show that culture of *stm* mutant explants in high CK conditions does not restore proper sustained shoot growth, supporting the idea of STM having CK-independent roles in meristem function. Furthermore, we show that *STM* is required for continued stem cell function in the SAM by sustaining expression of the stem cell-promoting factor *WUS* and preventing cells of the meristem organizing center from adopting lateral organ-specific fates. We also demonstrate that transcriptional activation of class-1 *KNOX* genes by STM is independent of *AS1*, since *AS1* transcript levels are not reduced in response to STM and STM is able to transactivate expression of both *KNAT1/BP* and *KNAT2* in the *as1* mutant background.

Shoot growth in higher plants is dependent on the activity of the shoot apical meristem (SAM). The SAM comprises a small, dome-shaped population of undifferentiated cells used for the formation of new lateral organs such as leaves at the meristem flank and as self-renewing stem cells in the meristem center. The *Arabidopsis* *SHOOT MERISTEMLESS* (*STM*) gene is expressed throughout the SAM, but not in lateral organ primordia, and is required for development of the SAM during embryogenesis and its subsequent maintenance throughout the plant life-cycle.^1^^-^^3^ *STM* is a member of the class-1 *KNOX* gene family and encodes a homeodomain transcription factor.^4^ Other class-1 *KNOX* genes include *KNAT1*/ *BREVIPEDICELLUS* (*BP*), *KNAT2* and *KNAT6*, and these are expressed in the SAM but excluded from leaf primordia where they are repressed by the MYB-domain transcriptional repressor ASYMMETRIC LEAVES1 (AS1).^5^^,^^6^

## 

### Cytokinin cannot replace STM function in the SAM

The *stm* loss-of-function mutants exhibit defects in SAM formation and maintenance. In strong alleles such as *stm-1*, the SAM does not form and cotyledons become fused^1^ (Fig. 1B). Since the SAM is not formed, leaves are not normally produced at the shoot apex. However, in milder alleles such as *stm-2*, the meristem is formed but is not properly organized.^2^^,^^3^ Organ primordia are initiated inappropriately resulting in irregular phyllotaxis and the eventual consumption of the cells of the SAM into organ primordia. As a consequence, the SAM is not maintained indefinitely, leading to shoot growth arrest. After meristem termination, new shoots are initiated in the axils of leaves before these too cease growth, resulting in an ‘abort-retry’ mode of shoot growth which can also be observed in those *stm-1* plants that are occasionally able to produce leaves (Fig. 1C). We find that similar *stm*-like phenotypes can be recapitulated using STM-specific RNAi (Fig. 1D-F), demonstrating that such defects arise through a post-embryonic requirement for continuous STM function. Overall these observations suggest that *STM* is required for the initial formation of the SAM and for its continued maintenance through proper organization mediated by restricting the formation of organ primordia.

**Figure:**
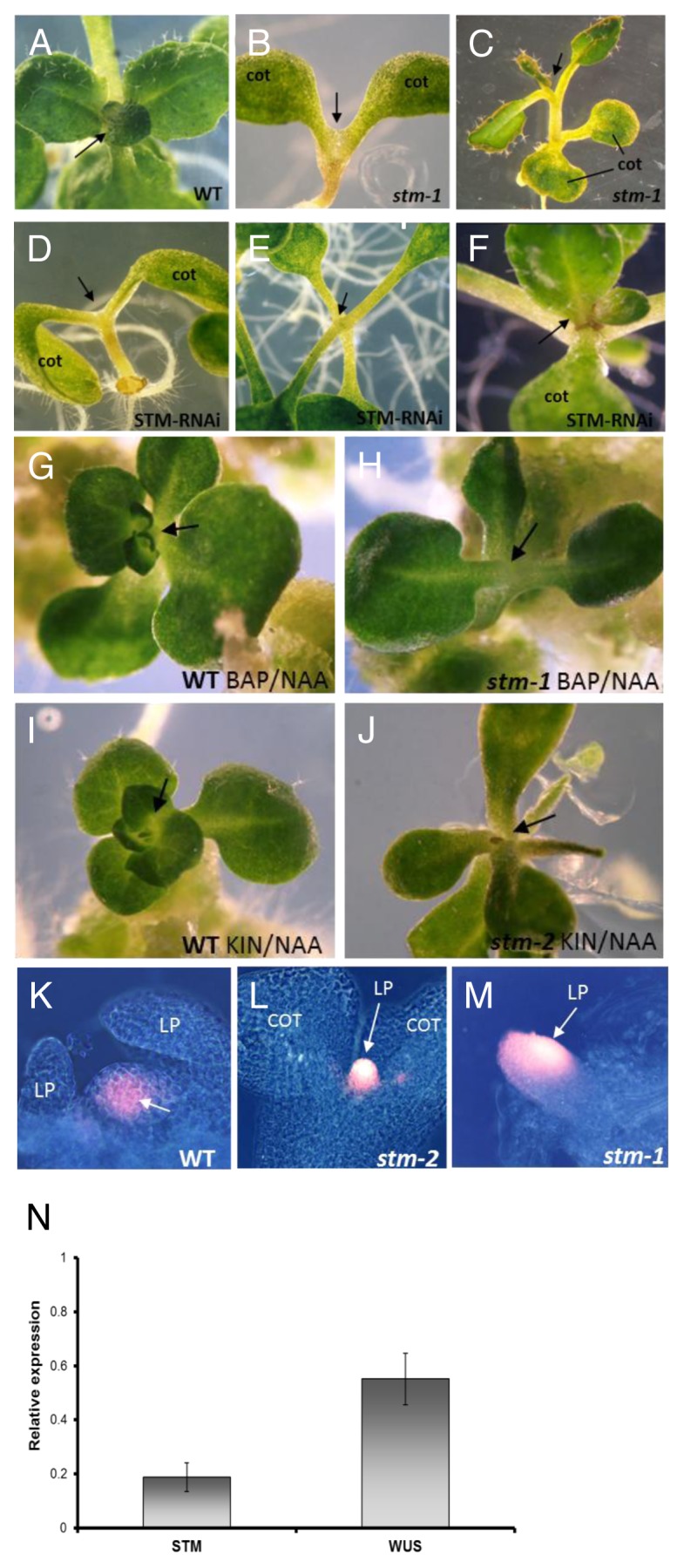
**Figure 1.** STM is required to maintain *WUS* expression and its function in SAM organization cannot be replaced by cytokinin. (**A**) WT shoot apex showing emerging leaves (arrow). (**B**) *stm-1* seedling with fused cotyledons and no emerging leaves (arrow). (**C**) *stm-1* mutant that has produced few adventitious leaves from the apex before terminating shoot growth (arrow). (**D**) STM-RNAi seedling showing lack of emerging leaves at shoot apex (arrow). (**E**) STM-RNAi seedling showing arrested shoot apex after the formation of 2 leaves (arrow). (**F**) STM-RNAi seedling showing shoot arrest with the formation of terminal leaf (arrow). (**G**) WT (Ler) shoot regenerated with 1000ng/L benzylaminopurine (BAP) and 300 ng/L napthyl acetic acid (NAA). Leaves emerge in regular spiral phyllotaxis (arrow). (**H**) *stm-1* shoot regenerated under the same conditions as (**G**). Arrow shows arrested shoot with no new emerging leaves. (**I**) WT (Ler) shoot regenerated with 1000ng/L kinetin and 300 ng/L napthyl acetic acid (NAA). Leaves emerge in regular spiral phyllotaxis (arrow). (**J**) *stm-1* shoot regenerated under the same conditions as (**I**). Arrow shows disorganized shoot without proper phyllotaxis that will terminate growth. (**K**) pWUS:GUS expression in the WT SAM. GUS activity is localized to the organizing center of the meristem (arrow) in a slightly more diffuse pattern than mRNA in situ hybridization studies.^7^ (**L**) pWUS:GUS expression in the *stm-2* mutant. Residual GUS activity is detected in a young emerging leaf (arrow). (**M**). pWUS:GUS expression in the *stm-1* mutant. Residual GUS activity is detected in a leaf primordium (arrow). (**N**) Real-time qRT-PCR analysis of WUS mRNA levels following induction of STM-RNAi for 72 h relative to induced L*er* (transgenic empty vector line). *WUS* transcripts are reduced to ~50% WT level. Data are averages from at least 3 experiments. Cot = cotyledon, LP = leaf primordium. Color inverted on GUS images (**K-M**). For shoot regeneration explants were cultured with various cytokinin (BAP or Kinetin at 300 ng/L – 3000ng/L) and auxin (NAA or 2–4D 100ng/L – 1000ng/L) concentrations with no organized, sustained shoot growth observed for *stm* explants under any of these conditions.

Previous studies have shown that STM induces cytokinin (CK) synthesis through induction of *ISOPENTYL TRANSFERASE* (*IPT*) gene expression, and that application of exogenous CK or targeted expression of *IPT* genes in the SAM partially restores meristem activity to *stm* mutants.^8^^,^^9^ However, proper phyllotaxis and sustained meristem function were apparently not restored under these conditions, suggesting that *STM* has additional meristem-organisational roles that cannot be replaced by CK.

We investigated if CK can provide SAM organization and sustained function independently of *STM* by regenerating shoots from *stm* explants under high CK conditions. Shoot formation was stimulated from calli derived from WT and *stm (stm-1 or stm-2)* root explants using various concentrations of CK (BAP or kinetin – see Fig. 1 legend) and auxin, and the morphology of shoots was compared. Shoots regenerated from WT (Ler) root explants showed regular phyllotaxis and sustained function (Fig. 1G, I), and eventually produced flowers. However, regardless of the presence of high concentrations of exogenous CK in the media, shoots regenerated from *stm* root explants consistently recapitulated the *stm* mutant phenotype, and despite forming a few leaves, these terminated in the same abort-retry manner observed in *stm* mutant plants that developed from seed (Fig. 1H, J). The failure of CK to restore normal meristem function was apparent in shoots regenerated from root callus of both *stm-1* and *stm-2* alleles, as well as in shoots regenerated from a transgenic STM-RNAi line, which displays a similar phenotype to *stm-2* (Fig. 1D-F; not shown).

We conclude that CK cannot fully replace STM function in the proper formation and maintenance of the SAM, even in tissue culture under a broad range of CK concentrations, supporting the results of Endrizzi et al.^2^ From this perspective, amelioration of the *stm* phenotype by increasing CK levels observed in other studies^8^ is likely due to an increase in the size of the cellular pool available for organ formation, since CK promotes cell proliferation through the CYCD3 pathway,^10^ rather than promoting meristem organization per se. This further supports the hypothesis presented in Scofield et al.^10^ that the role of STM in meristem development is not only to promote synthesis of CK, but also to promote meristem organization in a manner that is not replaceable by CK.

### STM is Required for Maintenance of WUS Expression

Previous studies have shown that when *STM* activity is lost, organ primordia form within the central zone of stem cells, suggesting a loss of stem cell identity.^2^^,^^3^^,^^10^ A key factor in controlling stem cell identity in the SAM is the *WUSCHEL* (*WUS*) gene, which encodes a WOX homeodomain transcription factor essential for stem cell maintenance.^7^^,^^11^ *WUS* is expressed in the meristem organizing center (OC) and promotes CK responses by repressing A-type *ARR* gene expression.^12^ These are primary CK response genes that act to negatively feedback on CK responses. Hence, while STM promotes CK synthesis in the SAM, WUS promotes CK responses. In agreement with *STM* and *WUS* having differing roles in the SAM, neither gene can replace the function of the other when ectopically expressed.^13^ *WUS* expression precedes *STM* in embryonic SAM development, demonstrating that STM is not required for the initial activation of *WUS* expression.^11^ However, induction of de novo meristem formation through ectopic expression of *STM* leads to activation of *WUS* expression and WUS function is required to maintain stem cell identity in these meristems.^10^ We therefore investigated whether *STM* is required to sustain *WUS* expression in the SAM.

We analyzed *WUS* transcript levels by qRT-PCR following short-term downregulation of STM by DEX-inducible RNAi, since the morphological meristem defects and variable re-initiation of shoot growth in *stm* mutants prevent meaningful comparative analysis of *WUS* transcript levels between *stm* and WT. We found *WUS* to be consistently downregulated, with transcript levels ranging from ~40% to ~60% WT level 72h after induction of STM-RNAi (Fig. 1N). The decrease in *WUS* expression was detectable before any morphological changes in shoot development associated with loss of *STM* were apparent, suggesting it is not merely a consequence of loss of meristem cells. *WUS* transcript levels were not significantly affected by short-term upregulation of *STM* (not shown), suggesting the regulation of *WUS* by STM might be indirect or that STM can only regulate *WUS* in certain cell types, consistent with its expression in only a subset of *STM*-expressing cells in the SAM. We therefore suggest that although *STM* is not required for the initial activation of *WUS* expression during embryogenesis, it is required to maintain *WUS* expression in the SAM.

To demonstrate that *STM* is required to maintain the integrity of WUS-expression domain (the OC), we examined expression of a pWUS:GUS reporter in *stm-1* and *stm-2* apices after termination of the SAM. In WT seedlings, GUS activity was detected in the OC in the SAM interior (Fig. 1K). In most *stm* seedlings, GUS activity was not detected after meristem termination, but in a subset we could detect residual GUS activity in terminal leaf primordia and young leaves (Fig. 1L, M). This demonstrates that the *WUS*-expressing cells of the SAM are incorporated into primordia in the absence of *STM*, further demonstrating that *STM* is required to maintain organizing center/ stem cell integrity.

### STM Regulates KNOX Genes Independently of AS1

The class-1 *KNOX* genes *KNAT1*/*BREVIPEDICELLUS* (*BP*) and *KNAT2* are closely related to *STM*^4^ and are repressed in leaf primordia by the MYB-family transcriptional repressor *ASYMMETRIC LEAVES1* (*AS1*), a member of the so-called ARP group of proteins that negatively regulate *KNOX* gene expression in diverse plant species.^5^^,^^6^ Previous studies have suggested that in the SAM, STM represses *AS1* expression, which permits expression of *KNAT1/BP* and *KNAT2*. This model is based on 2 observations. First, in the *stm* mutant embryo, *AS1* expression extends between cotyledon primordia into the region that would normally form the SAM, suggesting that STM could act as a repressor of AS1.^5^ However, this might also be a consequence of developmental epistasis, where the absence of SAM formation allows the domain of *AS1* expression to become continuous between cotyledons due to the adoption of a more differentiated leaf cell-like morphology. Second, Byrne et al.^6^ showed that in *stm as1* double-mutants, *KNAT1/BP* was able to functionally substitute for *STM*, and the authors proposed the regulatory model shown in Figure 2P. However, an alternative model in which STM and AS1 competitively regulate *KNAT1* has been suggested but not confirmed.^4^

**Figure:**
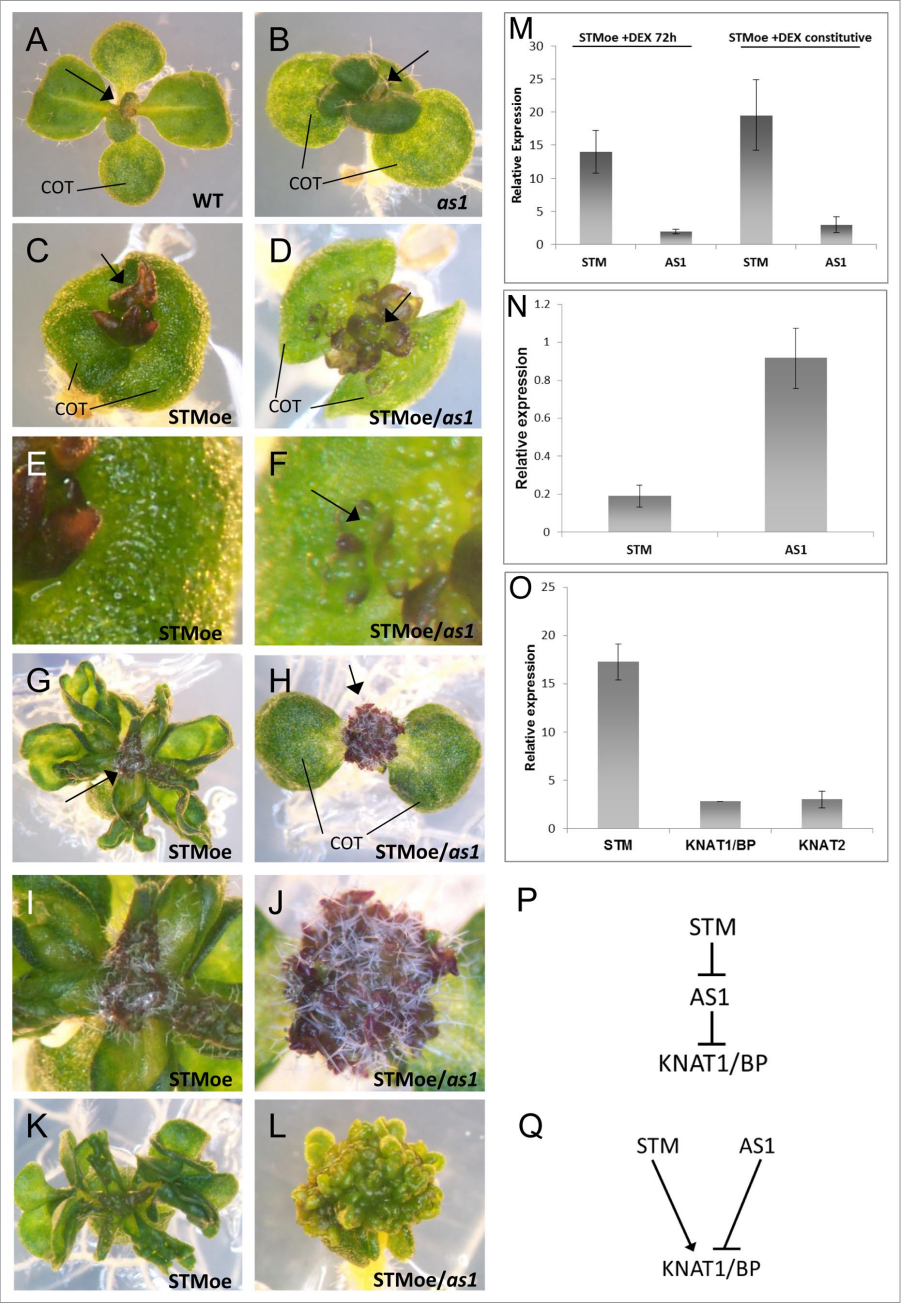
**Figure 2.** STM regulates *KNOX* genes independently of AS1. (**A**) WT seedling. (**B**) *as1* (*as1–1*) seedling. (**C**) STMoe seedling. (**D**) STMoe/*as1* seedling. Arrows indicate emerging leaves. (**E**) Higher magnification image of (**C**) showing no ectopic meristems on cotyledon surface. (**F**) Higher magnification image of (**D**) showing ectopic meristems of cotyledon surface (arrow). (**G**) STMoe seedling induced with DEX after germination (5 d after sowing; DAS). Arrow indicates emerging leaves. (**H**) STMoe/*as1* seedling induced 5 DAS. Leaf outgrowth is more severely inhibited than in (**G**; arrow). (**I**) and (**J**) Higher magnification images of (**G**) and (**H**) respectively. (**K**) STMoe plant in mid-vegetative growth induced from sowing. (**L**) STMoe/*as1* plant in mid-vegetative growth induced from sowing. Leaf outgrowth is reduced compared with (**K**). (**M**). Real-time qRT-PCR analysis of *STM* and *AS1* expression in STMoe plants induced with DEX for 72h or induced from sowing (constitutive) relative to induced WT empty vector control line. (**N**) Real-time qRT-PCR analysis of *STM* and *AS1* expression in RNAi plants induced with DEX for 72 h relative to induced WT empty vector control line. (**O**) Real-time qRT-PCR analysis of *STM*, *KNAT1/BP* and *KNAT2* expression in STMoe/*as1* plants induced for with DEX for 72 h relative to mock-induced plants. (**P**) Model for STM regulation of *KNAT1/BP* according to Byrne et al.^6^ (**Q**) Alternative model for STM regulation of *KNAT1/BP* (this study).

We analyzed *AS1* expression by qRT-PCR in response to induced upregulation of *STM* (DEX-inducible regulation) and consistently found no significant decrease in its expression level at any of the time-points tested, suggesting that STM does not repress *AS1* (Fig. 2M). Moreover, we detected an increase in *AS1* transcript levels following long-term induction of *STM* (up to 2-fold increase 72h after induction and ~3-fold increase in plants constitutively expressing *STM* from sowing). This increase in *AS1* levels might be attributable to feedback responses arising from long-term growth with high *KNOX* gene expression levels, rather than a direct response to *STM*. Likewise, we did not detect increased *AS1* transcript levels in STM-RNAi plants (Fig. 2N), again indicating that STM does not repress *AS1* expression.

We previously showed that STM promotes expression of both *KNAT1/BP* and *KNAT2*.^10^ To establish whether this regulation depends on an STM-dependent repression of *AS1* not detected in our qRT-PCR experiments, we induced STM upregulation in the *as1* mutant background and measured the transcript levels of *KNAT1/BP* and *KNAT2*. We found that *KNAT1/BP* and *KNAT2* were upregulated following transient STM induction in the *as1* background (approximately 3-fold change for both genes), and therefore reason that STM-dependent upregulation of these genes cannot be mediated through repression of *AS1* (Fig. 2O). If STM indeed repressed *AS1*, we would expect STMoe to repress *AS1* expression, resulting in little additional phenotypic enhancements in the STMoe/*as1* background. However, we observed a strongly enhanced phenotype in STMoe/*as1* compared with STMoe or *as1* alone suggesting that AS1 is indeed normally expressed in the STMoe line (Fig. 2A-L). We found that STMoe/*as1* seedlings showed enhanced capacity for ectopic meristem formation, especially on cotyledons where such meristems do not normally form in STMoe (Fig. 2F), and leaf outgrowth and differentiation were inhibited more strongly in STMoe/*as1* compared with STMoe (Figs. 2D, H, J, L). This could be attributable simply to higher levels of *KNAT1/BP* and *KNAT2* expression in STMoe/*as1* relative to STMoe, or could be due to enhanced *KNOX* gene sensitivity in the *as1* background, similar to the enhanced sensitivity to *KNOX* gene function conferred by expression of the cell cycle regulator *CYCLIND3;1*.^10^

These data strongly suggest that STM does not repress *AS1* and that regulation of *KNOX* genes by STM is AS1-independent. We therefore confirm the alternative model for the regulation of *KNOX* genes by STM and AS1, initially suggested in Scofield and Murray,^4^ in which STM and AS1 competitively regulate *KNAT1/BP* expression, though not necessarily directly (Fig. 2Q). This model explains that in the *stm* mutant, *KNAT1/BP* is not expressed because AS1 represses its transcription and the SAM does not form, while in the *stm as1* double mutant *KNAT1/BP* is de-repressed and can functionally substitute for *STM*. In normal development, *KNAT1/BP* is expressed in the SAM because AS1 is absent and is excluded from leaf primordia because STM is absent and AS1 is present. In STMoe plants, meanwhile, expression of *KNAT1/BP* occurs in leaves^10^ because STM is present in high levels and can alleviate the transcriptional repression by AS1. This model of regulation also applies to *KNAT2* but it does not provide meristem functions of *STM* or *KNAT1/BP*.

This study has addressed some of the key functions of STM and its regulatory interactions with other *KNOX* genes. We have shown that CK cannot replace STM function in meristem organization, demonstrating that STM has additional roles in the SAM apart from promoting CK synthesis. We also showed that while not essential for initial activation of *WUS*, STM is required for maintenance of *WUS* expression and the integrity of the organizing center and stem cell niche. Finally, our data support a refined model which suggests that STM and the MYB-domain transcriptional repressor AS1 competitively regulate *KNOX* gene expression.
